# Exploring refugees' experience of accessing dental health services in host countries: a scoping review

**DOI:** 10.3389/froh.2024.1328862

**Published:** 2024-03-12

**Authors:** Elaf Asfari, Andrea Rodriguez, Arek Dakessian, Siyang Yuan

**Affiliations:** ^1^ School of Dentistry, University of Dundee, Dundee, Scotland; ^2^ Institute for Global Health and Development, Queen Margaret University, Musselburgh, Scotland

**Keywords:** refugees, dental health services, oral health, host countries, dental health provision, accessibility

## Abstract

**Introduction:**

Refugees often face worse oral health outcomes, such as periodontal diseases and dental caries in host countries due to barriers including language and cultural differences, institutional discrimination, and restricted use of dental health services. This scoping review aims to map and summarise the available studies on refugees’ experience of accessing dental health services in the host countries, to identify the main characteristics of the dental health services that refugees access and to explore the barriers and enablers to navigate the dental health service system in their host countries.

**Methods:**

The Joanna Briggs Institute (JBI) framework was adopted. PubMed, Scopus, Assia, CINAHL and Social Services Abstract were searched. A search strategy was developed using Medical Subject Headings (MeSH) terms and a combination of search operators and syntax used in MEDLINE were adopted for the remaining databases. Data were synthesised using thematic analysis.

**Results:**

Fourteen articles were included. Most studies used qualitative methods and Australia seemed to be the country with the highest number of publications surrounding this topic. The included studies showed that refugees frequently encountered substantial obstacles when attempting to access dental services in host countries. Numerous barriers such as language barriers, cultural differences, and lack of health insurance or financial support hindered refugees' ability to access these services. Additionally, many refugees possessed limited knowledge of the dental care system in their new country. As a result of untreated dental problems, refugees suffered from pain and other health complications.

**Discussion:**

This scoping review explored the challenges refugees have experienced in accessing dental health services in host countries, which included the key barriers such as affordability, accessibility, accommodation, availability, awareness, and acceptability. The scarcity of relevant research highlighted the need for a more comprehensive understanding of refugees’ experiences accessing dental health services in host countries. Limited data were identified regarding evidence focusing on the characteristics of dental services accessed by refugees in host countries.

## 1 Introduction

A refugee is defined as someone who has been compelled to escape their home country due to violence, war, or persecution and who is unable to return home or is afraid to do so (1). The health of migrants is usually neglected in broader discussions on migration, and they are usually ignored as a population in health strategies (2).

### 1.1 General health of refugee groups in host countries

Refugees experienced mental health issues, as a result of all the persecution and conflicts that they were witnessing in their home country (3). Evidence from a systematic review in 2020 has shown nearly 1 in 3 refugees suffered from depression (4). In addition, the prevalence of anxiety disorders was estimated to be around 11% (4). This is in line with another study conducted in refugee camps indicating a high prevalence of probable post-traumatic stress disorder (83.4%) and depression (37.4%) among Syrian refugees residing in Turkey (5). Moreover, refugees have a high risk of physical, social, and psychological diseases due to their traumatic experiences and stressors related to their resettlement in the host country (4). A study by Rehr et al. showed that about 20% of Syrian refugees residing in northern Jordan suffer from at least one non-communicable disease with type 2 diabetes and hypertension prevailing as the most prevalent conditions (6). A qualitative study of Iraqi refugees in Sweden demonstrated that approximately 77% of the participants experienced a significant level of psychological distress. Additionally, 73% of the participants reported suffering from somatic symptoms and around 90% of them reported having experienced traumatic events (7).

### 1.2 Oral health of refugee groups in host countries

According to the World Health Organization (WHO), oral diseases are one of the major health issues that refugees face (8). Refugees have experienced a high burden of oral diseases, such as periodontal disease and dental caries (9). It was found that 80% of refugees in Canada had untreated caries or periodontal disease (10). Tooth decay is the primary cause of tooth extractions among refugees (11). These were the most common dental treatments resulting in a high level of missing teeth which has a great impact on their quality of life (12–14). This is in line with the evidence from New Zealand discovering limited use of preventive dental treatments and more problem-oriented dental visits such as tooth extractions (15). Many participants reported experiencing tooth pain, with just over 50% receiving only one oral hygiene advice session since arriving in the country. Additionally, 50% had at least one tooth extraction and approximately 30% had scale and polishes performed since arriving in Dunedin (15).

### 1.3 Access to dental health services

Migrants, refugees and asylum seekers often face multiple disadvantages during their stressful migration processes (16). Compared to the local population in host countries, refugees tend to have fewer regular dental visits (17). There are limited data that describe the oral health care access and oral health status of refugees, especially for those who came from regions badly affected by the war, such as the Middle East and Myanmar (18).

Refugees face financial hardship and may not prioritise oral health issues after arriving in the host country (19). Moreover, refugees face challenges in accessing healthcare services in transit countries, particularly when they are living in temporary settlements and refugee camps (9, 14). These difficulties include limited infrastructure and resources in these settings, poor knowledge about available services and language barriers (9, 14). Other barriers hinder their access to dental health care including anxiety or fear of dental treatment, high treatment cost, language barriers, long distance to the care and long waiting time (9).

The healthcare system in the host countries also affects asylum seekers and refugees' (ASRs) access to oral health care as the system varies from country to country (16). For example, in Finland and Sweden, ASRs can receive oral health care funded by the government after granting permanent resident status (16). In Canada, only people who have been recognised as refugees before arriving in the country can benefit from the government fund which is only for basic and emergency care in the first 12 months of arriving in the country (16). In Germany, access to dental health services is limited in the first 18 months of arrival (9). Refugees in Canada may face financial hardship when accessing dental care as dental care services are not publicly funded (20).

Many dental healthcare interventions have been implemented to overcome social exclusion in dental practice and to tackle inequality among refugees, however, a structural barrier still exists hindering those populations from accessing dental healthcare (21). Therefore, oral health inequality among refugees is a major public health concern that should be taken into consideration (21). In terms of social support, a study conducted in Germany showed that a lack of adequate social support was associated with a reduced likelihood of utilising regular dental check-ups among the migrant population (22).

Despite the established literature on refugees' experiences of accessing medical services, little is known about their access to dental health services (21). This scoping review is aimed at exploring the existing evidence of refugees' experience in accessing dental health services. Three specific objectives:

[a]. To map and summarise the available studies on refugees’ experience of accessing dental health services in the host countries.
[b]. To identify the main characteristics of the dental health services that refugees access.
[c]. To explore the barriers and enablers to navigate the dental health service system in their host countries.

## 2 Methods

This scoping review applied the framework of Joanna Briggs Institute (JBI) using Population, Concept and Context (PCC) to guide the development of the research question and the eligibility criteria as followed (23):

➢ Population: Adult refugees (aged 18 years or older).
➢ Concept: Refugees' experience of accessing dental health services.
➢ Context: Host countries.

### 2.1 Eligibility criteria

Based on the JBI approach, the inclusion and exclusion criteria were developed (Table 1) to help selecting appropriate papers (23). Child refugees have more complex dental care needs, and they often rely on parents/caregivers to access dental care (24). Given the complex circumstances for children, we decided not to include children in this review. Different study designs were included. Primary research such as quantitative, qualitative, and mixed-method studies, as well as secondary research, such as systematic reviews, and documentary analysis studies were included.

**Table 1:** Inclusion and exclusion criteria.

Criterion	Inclusion	Exclusion
Language	English	Non-English studies
Type of papers	All published primary or secondary research.	Papers that were not published. Papers that were discussions or opinion pieces, news, and stories.
Literature focus	• Studies which answer the literature objectives • Papers which focus exclusively on accessing dental services among refugees in their host countries.	Studies that did not cover the scoping objectives.

### 2.2 Search strategy

The search was conducted for studies published between 01/01/2000 and 30/05/2022. The year 2000 was chosen as a starting period due to the presence of a notable refugee crisis worldwide (25–28).

An initial search of MEDLINE (PubMed) was undertaken followed by an analysis of the text words contained in the title, the abstract and the index terms. In the second search, all identified keywords and index terms were used across all included databases. The third search, the reference list of all included articles was searched for additional studies. Studies published in the English language were considered for inclusion in this review. The databases that were searched included PubMed, Scopus, Assia, CINAHL and Social Services Abstract. Search terms are presented in (Table 2).

**Table 2:** Pubmed search strategy.

1.	Dental Care [MeSH]
2.	Dental Health Services [MeSH]
3.	Oral Health [MeSH]
4.	Dental Clinics [MeSH]
5.	Dental Caries [MeSH]
6.	Dental services
7.	Dental providers
8.	Dental health
9.	Dental treatment
10.	Oral health
11.	OR/1–10
12.	Refugees [MeSH]
13.	Refugees
14.	OR/12–13
15.	Delivery of Health Care [MeSH]
16.	Health Services Accessibility [MeSH]
17.	Access*
18.	Availab*
19.	Delivery
20.	Provision
21.	Provid*
22.	Host
23.	Equitable
24.	Equity
25.	OR/16–25
26.	11 and 15 and 26

**Figure 1:**
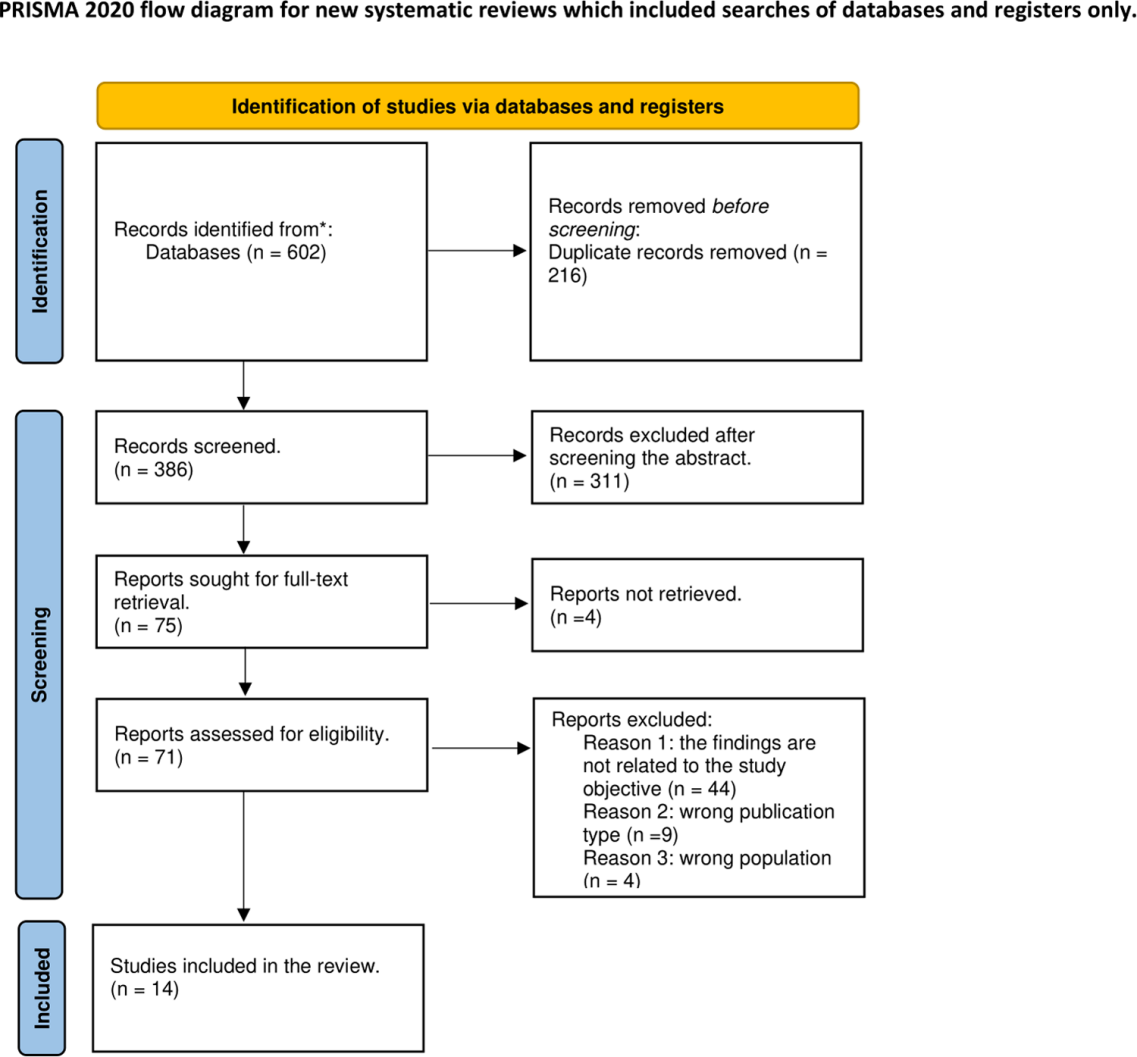
Prisma flow chart.

After conducting the initial search, all eligible articles were imported into Endnote X9.3.3. The titles and abstracts of these articles were reviewed using Rayyan. Full texts were then read by EA and SY, and reference lists were manually searched for any additional relevant papers. A flow chart was created following the guidelines outlined in PRISMA-ScR (Preferred Reporting Items for Systematic Reviews and Meta-Analyses: Scoping Review) (Figure 1).

### 2.3 Data charting and synthesis

A data extraction form was adapted from JBI to record key information relevant to the review questions. A narrative summary of the included studies was categorised based on the key parameters, such as authors, country of origin, aims, study description and key findings. All the selected articles were summarised (Table 3).

**Table 3:** Summary of all included studies.

Authors	Country of origin	Aims	Study description	Key findings
Davidson et al. (19)	Australia	to map public dental services across Australian jurisdictions, to identify barriers to accessing dental care, to identify gaps in provisions and to provide recommendations for improving access and oral health promotion for this group.	Data was requested from various sources: oral health care units, survivors of torture, auditors-general reports, the Australian Institute for Health and Welfare, and the University of Adelaide's dental statistics unit.	Refugees in Australia, especially in rural and regional areas, continue to face limited access to dental services. Numerous barriers, such as long wait times, inconsistent assessment criteria, varied eligibility requirements, and insufficient interpreter services, deter refugees from utilizing these services.
Due et al. (29)	Australia	To improve understanding of Middle Eastern refugees’ and asylum seekers’ oral health help-seeking and to determine the utility of Andersen's Model in this context.	Interviews were made with refugees and asylum seekers, and dental health practitioners.	Several participants indicated that barriers, such as transportation, long waiting times, and financial resources impede oral health assistance seeking. Additionally, uncertainty about eligibility, systemic healthcare factors, and past experiences impact future help-seeking behaviour.
Ay et al. (30)	Jordan	to identify the perceived health care service needs, accessibility of services, and barriers to access among a group of Syrian refugees living outside the camps in four governorates of Jordan and to compare the differences in access among them	A structured questionnaire was incorporated with a total of 196 completed surveys	Dental services are essential for maintaining oral health, as 43% of participants found access to them positive, and 62% of those needing them found it accessible.
Keboa et al. (16)	Global	to summarize and describe the prevalence of oral diseases, to describe access to and utilization of dental services and to describe extant strategies to improve oral health of this population.	The revised Arksey and O’Malley methodological framework was adopted for this scoping review, and a quality assessment was included. (CASP)	The study found that refugees in host countries have limited access to oral health care, influenced by factors such as healthcare systems, social structure, socio-economic status, culture, and personal oral health beliefs, resulting in longer treatment completion times.
Keboa et al. (31)	Canada	To understand the oral healthcare experiences of humanitarian migrants in Montreal and their perceptions of ways to improve access to oral healthcare.	The study used focused ethnography and the McGill Illness Narrative Interview (MINI) to interview humanitarian migrants in Montreal who received or required dental care.	Participants in Canada found dental care accessible, but some sought advice abroad due to financial constraints and high treatment costs. Cultural perceptions and missed appointments impacted the dental care process. Despite language barriers, dental care quality was excellent, and participants appreciated the kindness of dentists.
Kidane et al. (9)	Germany	to explore the access of Eritrean refugees and asylum-seekers to oral health care services in Heidelberg, Germany, as well as their perceptions and attitudes towards oral health care	The use of online semi-structured interviews was implemented.	The study revealed challenges in accessing dental services and navigating the German health system, with some participants expressing dissatisfaction with treatment decisions, perceived bureaucratic hurdles, communication barriers, cost and fear of migration impacting dental care utilization.
Nurelhuda et al. (20)	Canada	To explore the dental needs and access to dental services among migrants in Ontario, Canada	A brief questionnaire and semi-structured interviews were conducted.	Participants expressed dissatisfaction with the Interim Federal Health Program's benefits, including long waiting times, language barriers, and difficulty tracking appointments. Some received information from newcomer organizations, but others felt unsatisfied. Some self-treated due to high costs, while others received incomplete treatment. Many considered seeking care abroad in the future.
Okunseri et al. (32)	US	To describe the SROH, self-rated general health (SRGH), and use of dental/physician services; and to identify the factors associated with self-rated oral health (SROH) among Hmong adults.	A cross-sectional study design was implemented using locating sampling methodology. An oral health questionnaire was utilized to evaluate SROH and SRGH.	39% of participants did not have a consistent dental care provider, while 46% rated their access to dental care as poor or fair. 43% had visited a dentist within the past year. The majority (81%) had dental insurance. Among those who visited a dentist, 47% did so for a regular check-up. Having good or excellent self-rated oral health was linked to having dental insurance
Paisi et al. (33)	Host countries of very high development.	to identify the barriers and enablers to dental care access for ASRs in host countries of very high development.	Only qualitative research studies were incorporated.	Healthcare professionals face limited availability and awareness of services for refugee pregnant women, leading to false charges and restricted referring and utilisation. Inconsistent messages, dental anxiety, miscommunication, and differing expectations hinder access to care. Cultural attitudes, male influence, and policy differences impact access to dental treatment, with concerns about exclusionary dental benefits for ASRs and permanent residents.
Riggs et al. (18)	Australia	to describe Afghan and Sri Lankan women's knowledge and beliefs surrounding maternal oral health, barriers to accessing dental care during pregnancy, and to present the perspectives of maternity and dental service providers in relation to dental care for pregnant women.	Focus groups were carried out with participants of Afghan and Sri Lankan refugee backgrounds, as well as with midwives and dental service staff.	The study highlights barriers faced by non-immigrant pregnant women and refugees/asylum seekers in accessing dental care, including lack of awareness about “priority access” policy, limited understanding of maternal oral health, confusion about dental attendance, cost, and long waiting times.
Saadeh et al. (34)	US	This study evaluated the self-reported oral health status, practices, and access to care of adult refugees living in San Antonio, Texas, United States.	A cross-sectional study was conducted using a self-report survey to gather data from war-affected refugees who have resettled in San Antonio.	The study reveals that a significant number of participants face barriers to dental care, with 52% unable to access it in the past year. Financial constraints, such as lack of insurance and funds, were common reasons. The pain was the primary driver of dental visits, with 43% not visiting in over five years. Acculturation, particularly for refugees who recently moved to the US, significantly influenced access to care, with 90% citing insurance or affordability issues as the main reasons.
Eiad Zinah and Heba M. Al-Ibrahim (13)	Europe	To explore the status of oral health among refugees and asylum seekers groups I Europe by examining the available literature and to determine which evidence exists regarding the problems they face in terms of oral health.	The revised Arksey and O’Malley framework was adopted for this scoping review.	Refugees in Europe face challenges in accessing dental care due to financial constraints, treatment anxiety, language barriers, cultural differences, and unfamiliarity with healthcare services. Limited dental professionals and migration can disrupt their utilization of dental services.
Kang et al. (35)	UK	To examine ASR experiences accessing primary health care in the UK in 2018.	Face-to-face semi-structured recorded interviews	Participants reported receiving inappropriate dental treatment due to lack of interpretation and confusion between dental services, emergency care, and general medical practices. Some received free dental treatment through the low-income scheme, but faced bureaucratic application and charges. The shortage of NHS dentists and lack of awareness further complicated the situation.
Sypek et al. (36)	Australia	To explore the reported impact of regional resettlement of refugees on rural health services and identify critical health infrastructure for refugee's resettlement.	A comparative case study was conducted, using interviews and situational analysis.	The inadequate public dental services in towns led to refugees seeking private dental care, with some resorting to funding or seeking treatment in Sydney.

## 3 Results

A total number of 602 articles, including duplicates, were retrieved from the five databases. There were 386 articles selected for screening after removing 216 duplicates with Endnote X9.3.3 software. A total of 311 articles were then excluded after screening for relevant titles and abstracts. The full text of 4 articles could not be retrieved, which left a total of 71 papers for full-text review, of which 14 were finally included in this review based on the selection criteria. Details are provided in the PRISMA-ScR flow diagram below (Figure 1).

### 3.1 Study characteristics

The findings explored refugees' experience of accessing dental health services in their host countries. The key findings are organized based on the year of publication, country of origin, study design, the characteristics of the dental health services, and barriers and enablers of accessing these services.

Many of the included studies were published after 2016. This could be due to the increased influx of refugees resulting from various conflicts and wars in countries such as Syria. Half of the studies were qualitative (9, 18, 20, 29, 31, 35) with two interviews with refugees (9, 20) and one being an ethnographic study (31). Two cross sectional studies (32, 34) and two scoping review studies (13, 16), one systematic review (33) and one documentary analysis were also included (19). Most of the studies based were conducted in Australia (*n* = 4) (18, 19, 29, 36) followed by the United States (*n* = 2) (32, 34) and Canada (*n* = 2) (20, 31).

### 3.2 The characteristics of dental health services

The evidence shown that refugees in their host countries can access various types of dental services: dental clinics, which are usually located in easy-to-reach areas like shopping centres and near public transport (9, 13, 31), private clinics (31), government dental clinics (36), community clinics supported by local organisations (31, 33), and university dental clinics that offering a wide range of treatments (31). Hospitals were also mentioned in three studies as a service that can offer dental care for refugees at reduced costs (13, 31, 36).

Other included studies mentioned the use of mobile dental units that deliver basic dental care in vans that go to underprivileged areas (16, 31, 33), emergency dental care (16) with after-hours services/phone support (36), community dental programs offering a spectrum of services including emergency and general dental treatment (36), and community organisations that alongside their support activities provide dental services to refugees (13, 16, 31).

### 3.3 Experience of accessing dental health services in host countries

Six dimensions including affordability, accessibility, accommodation, availability, awareness, and acceptability were identified to synthesise the findings of this scoping review on the barriers and facilitators that refugees experienced when accessing dental health services. These dimensions were echoed in a systematic review as one of the included studies in this scoping review (33). For example, affordability was assessed by examining the financial burden of dental care on refugees. Accessibility was evaluated by considering factors such as the distance to dental clinics. Accommodation was measured by assessing the availability of interpreters, long waiting lists and delayed treatments. Availability was evaluated by examining the number of dental providers in the area, and the range of services offered. Awareness was measured through refugees' understanding of how to access the services and the collaborations between healthcare professionals. Lastly, acceptability was evaluated through cultural influences, refugees’ oral health beliefs and communication with dental health providers (33).

#### 3.3.1 Barriers

##### 3.3.1.1 Affordability

Affordability significantly impacts the experiences of refugees accessing dental health services, as they often encounter challenges due to the high cost of treatments, transportation expenses, and limited financial resources (9, 16, 18, 20, 29, 31, 33, 34, 36).

Lack of dental insurance is one of the major barriers faced by refugees when accessing dental health services in their host countries (16, 34). The high cost of dental treatment and travel expenses to access the dental clinic are some of the most common obstacles faced by refugees (9, 16, 18, 20, 29, 31, 33, 34, 36). In addition to the high treatment related cost, the low income of the refugees serves as other impediments to accessing dental health services and has also prevented some of them from completing the dental treatment (20). Refugees are not eligible for the same dental benefits as the native population, which can limit their access to dental health services and can result in a high prevalence of dental problems (33).

##### 3.3.1.2 Accessibility

Accessibility plays a crucial role in the experiences of refugees accessing dental health services, as they frequently face obstacles related to geographic location, limited information availability, and extended waiting periods for treatment (9, 16, 18, 19, 31, 33, 36). One of the main obstacles to receiving dental treatment among refugees was obtaining trustworthy information about health care services or someone who could guide them to navigate the health care system (9, 18). Moreover, community workers who were responsible for supporting refugees suffer from heavy workloads which made them de-prioritise the dental problem (33).

Some included studies demonstrated that mobility issues and long-distances are significant barriers in accessing dental health services (9, 33, 36). Weather conditions in some host countries can also influence refugees' decisions in attending the dental appointments (31, 33).

The lengthy period to receive treatment is another major concern (16, 19, 29). Long waiting time to receive dental treatment in the host countries could lead to dissatisfaction, making refugees unwilling to access dental health services (31, 33).

##### 3.3.1.3 Accommodation

Accommodation emerges as a significant barrier for refugees seeking dental health services, as the non-availability of interpreters and language barriers hinder effective communication with healthcare providers, leading to difficulties in explaining dental problems, perceiving inappropriate treatment, and building provider-patient relationships (9, 13, 16, 18–20, 33–35). The non-availability of the interpreters was highlighted as a major significant barrier when accessing dental health services by many studies (9, 13, 16, 18–20, 33–35). The absence of interpreters in the host countries can lead to perceiving inappropriate dental treatment as patients might find it difficult to explain their problems (33). Dependency on interpreters and the issue of confidentiality were also mentioned as a hindrance by Eritrean refugees in Germany in another study (9).

Some studies demonstrated that refugees faced difficulties in building provider-patient relationships resulting in reduced access to appropriate dental health services (9, 18, 31, 33, 34). Without English skills, refugees will be unable to interact with their healthcare providers and therefore unable to build a trusting relationship with their providers (9, 18, 31, 33, 34).

##### 3.3.1.4 Availability

The limited availability of dental health providers and scarcity of public dental services pose significant challenges for refugees accessing comprehensive dental treatment. Due to the shortage of dental health providers in most of the host countries in Europe, dental treatment has been limited to just tooth extraction in the refugee camps (13, 35). Moreover, the scarcity of public dental services has also been reported in some studies (31, 36).

##### 3.3.1.5 Awareness

Lack of awareness among refugees regarding the functioning of the healthcare system, accessing healthcare services, bureaucratic hurdles, and limited collaboration and communication between healthcare sectors can pose significant obstacles to refugees' access to dental health services (13, 18, 29, 31, 33, 35).

Poor knowledge in understanding of how the healthcare system functions in the host countries and how to access the healthcare by refugees can be significant obstacles to accessing dental health services (13, 18, 29, 31, 33, 35). The bureaucracy associated with accessing the health care system was also mentioned by some refugees in the UK and Germany as a hindrance to accessing the services (9, 35). Lack of collaboration and communication between healthcare providers working in different healthcare sectors can also have negative effects on refugees' access to the dental health services (18, 31, 33).

##### 3.3.1.6 Acceptability

Acceptability presents significant challenges for refugees accessing dental health services, as cultural differences, experiences of hardship during migration, fear of dental treatments, negative perceptions, and previous negative experiences can all contribute to barriers in seeking dental care, including feelings of humiliation, misunderstanding, and receiving inappropriate treatment from healthcare providers (9, 13, 16, 18, 33, 36). Four studies have shown that cultural differences can pose an additional obstacle to accessing dental health services (9, 13, 31, 33). Furthermore, the severe hardship that refugees endured during the migration route and the integration into a new culture have a disruptive impact on the use of dental services (9, 13).

Fear of dental treatments and apprehension of the dental instruments was considerable as barriers to seeking dental care among refugees in the host countries (9, 13, 18, 33). Refugees' beliefs and perceptions about oral healthcare and their previous dental care experience also affect their access to dental health services (9, 13, 16, 18, 33, 36). Moreover, the expected time to finish the dental treatment is longer for this group compared to the nationals (16). In one of the included studies, some participants reported that they felt humiliated, provided with inappropriate dental treatment, and misunderstood by healthcare providers (31).

#### 3.3.2 Enablers

Despite these challenges, the review has identified certain factors that facilitate refugees' access to dental services in some countries. These factors include positive relationships between refugees and their dentists (9, 31), the accessibility of dental services in some countries for refugees (30, 31). Moreover, medical coverage that assists refugees in accessing services in some nations in the first few months (34). Language barriers do not appear to significantly impede access to dental health services in Canada (31). However, some refugees prefer consulting with dentists who speak their native language (31).

Health coverage for refugees varies across countries. For instance, in the US, refugees receive medical coverage that enables them to access dental health services for the first eight months of arriving the country. However, they begin to experience difficulties once the coverage ends (34). Two studies also suggest that the relationship between refugees and their dentists is generally positive, with many refugees expressing satisfaction with the quality of care they receive and the compassion and dignity with which they are treated (9, 31). A study examining healthcare service needs, service accessibility, and barriers to access among Syrian refugees residing outside camps in Jordan demonstrated that approximately 60% of participants reported accessible dental clinics (18, 30). Although numerous obstacles remain, these enablers present promising solutions for enhancing refugees' access to dental services.

## 4 Discussion

Refugees face significant challenges in accessing dental health services in their host countries. This scoping review has identified several barriers to accessing these services (9, 16, 18, 20, 29, 31, 33, 34, 36). Despite these challenges, the scoping review has also highlighted some factors that make it easier for refugees to access dental health services in some countries. These include positive relationships between refugees and their dentists and medical coverage that helps refugees to navigate dental health services in some countries (9, 31).

This scoping review alone is insufficient to address the three objectives of the study. It has briefly mentioned the type of dental services available for refugees which include dental clinics, hospitals, mobile dental clinics, emergency dental care, community dental programme and community organisations (9, 13, 31, 33, 36). This indicates a potential research gap in certain aspects of refugees’ access to dental health services, which could be an area for future study. According to a global survey conducted by Kateeb et al. to explore the current policies and interventions related to refugee oral health found that emergency dental services are the primary oral health services provided to refugees upon their arrival in host countries, followed by therapeutic and preventive care (37). However, this study identified that these services are not sufficient to meet the oral health needs of refugees (37).

Most of the included studies were from developed countries with Australia having the highest number of studies based on geographic location (18, 19, 29, 36). Furthermore, only one study has focused on refugees' experience in accessing dental health services within refugee camps (13). As a result, further research is required in these areas. Research by Kateeb et al. suggested that a collaboration between National Dental Associations, international organizations, and other health agencies working in refugee camps is crucial (37). Furthermore, the study emphasizes the importance of implementing promotional oral health programs in refugee settings (37).

Many of the included studies were published after 2016. This could be due to the increased influx of refugees resulting from various conflicts and wars in countries such as Syria. This heightened the need for research on the topic of refugees' experiences accessing dental services which may have led to increased funding for research in recent years, contributing to the rise in publications. Most of the included studies were qualitative (9, 18, 20, 29, 31, 35). This reflects the increasing awareness over the last few years of the importance of using qualitative designs as a way of exploring in-depth aspects that are not captured through quantitative methodologies.

The findings will be discussed under the following themes.

### 4.1 Affordability

The high cost of dental treatments, coupled with transportation expenses to visit dental care facilities, poses considerable challenges for refugees seeking these services (9, 16, 18, 20, 29, 31, 33, 34, 36). This finding aligns with previous research, which has shown that many refugees lack dental insurance or face financial constraints that hinder their access to dental care (38, 39).

Furthermore, the availability of dental health insurance for refugees varies greatly between countries and even within the same nation, due to the diverse healthcare system policies across national and regional contexts (16). It is crucial to address these disparities and develop more inclusive policies that ensure equitable access to dental health services for refugee populations. By doing so, we can work towards improving the overall health and well-being of these vulnerable communities, who often face numerous challenges in their journey to rebuild their lives.

### 4.2 Accessibility

Accessibility is a critical factor in understanding refugees' experiences with dental health services, as they often encounter challenges related to geographic location, information availability, and lengthy waiting times for treatment (9, 16, 18–20, 29, 33). These issues have been also emphasized in the literature (39)*.* Flexibility in healthcare services, such as relocating services to more accessible areas, adapting working patterns to suit refugees' needs, and providing specialized services, has also been recommended in the literature (40).

Four studies from this review recognised challenges associated with the extensive workload of organizations supporting refugees, which often leads health or social care workers to deprioritize dental health issues (18, 20, 29, 33). These findings were also mentioned in the wider literature (40).

A previous study has shown that improving access to dental health services can be achieved through cooperation between different healthcare services. By identifying these services and potential areas of collaboration through referral pathways and joint service delivery, both health providers and service users can benefit (40). This underscores the importance of refugee organizations and other stakeholders and cooperation between other health services in guiding refugees through the healthcare system and helping them understand the appropriate health coverage for their needs.

### 4.3 Accommodation

Language barriers and communication issues with healthcare providers also pose significant challenges (9, 13, 16, 18–20, 33–35). A previous study has shown that refugees often rely on their children or other family members to help them remember appointments and understand and communicate with healthcare providers, resulting in inappropriate translation and hence risks to patient safety (38, 39). Furthermore, language barriers have limited their ability to understand and follow medical instructions and impacted their relationships with healthcare providers.

Many studies in this scoping review highlight the challenges refugees face in accessing dental health services due to difficulties in building dental provider-doctor-patient relationships and communication barriers (9, 18, 31, 33, 34). Two of the studies from the literature indicated that refugees expressed their gratitude for the care offered and appreciated the expertise of the health providers (38, 40). However, prior research has indicated that even with a strong relationship between healthcare providers and refugees, the transient nature of some users complicates the development of long-term trust with healthcare professionals (40).

This study has not highlighted the impact of illiteracy and limited knowledge of the local language. Shame and isolation were other barriers mentioned in previous studies (39). Educating healthcare professionals to provide culturally appropriate and patient-centred care is highly important and requesting trained interpreters to improve effective communication between refugees and health providers can improve their access (39).

### 4.4 Availability

The scarcity of healthcare providers in some areas was also a significant problem for refugees mentioned in this study (13, 31, 33, 35, 36). This finding aligns with previous research indicating a lack of specialist services in rural regions which in turn puts additional efforts and workload on healthcare professionals (38). Therefore, implementing strategies and plans to expand the staff capacity across different geographic locations to enhance access to dental services in underserved neighbourhoods is crucial.

### 4.5 Awareness

This review recognised challenges related to inter-professional collaborations among healthcare providers and refugees’ awareness in accessing dental health services particularly when healthcare pathways were unclear or inconsistent (18). These elements were also highlighted in the wider literature (38, 41).

Previous research demonstrated that complex referral pathways have been found to impede service access among refugees (38). This finding is consistent with a study from the current review conducted in Australia, which revealed a lack of cooperation between healthcare providers when referring patients within the healthcare system (18).

An effective referral system would increase refugees' awareness of available services and would facilitate their access to these services (40). Moreover, raising social workers' awareness of the importance of assisting refugees in accessing dental services is of paramount importance. This should be taken into consideration when designing and renewing the health care system in the host countries and should also be a hot topic for future research.

### 4.6 Acceptability

Five studies in this review mentioned that cultural differences can pose additional barriers to accessing dental services (9, 13, 31, 33, 34). Previous research reported that health providers do not understand refugees past traumatic experiences, and they face difficulties in discussing sensitive issues with refugees. However, other studies mentioned the positive attitude and caring connection between healthcare providers and refugees, noting that providers respect their culture which helped them access the services (38, 40).

The issue of confidentiality between refugees and healthcare providers has not been clearly addressed in the findings of this scoping review. A study from the literature discovered that some refugees fear their information might be shared with immigration authorities. To overcome these challenges, healthcare professionals have found it crucial to clearly explain their role and assure patients that their information will remain confidential. This approach can help build trust and alleviate suspicions (38, 40).

This scoping review did not find any significant findings related to health literacy. Health literacy was another barrier mentioned in the literature. Many refugees struggle to understand certain terms used in the healthcare system, such as self-management and preventative care (e.g., screening) (40).

Previous literature showed that recognising the cultural differences of refugees is a vital enabler to improve their access to health services. Moreover, healthcare providers believe that specific training would improve their confidence and facilitate their clinical practice (40). This understanding includes interpreting body language and acknowledging value differences, which helps healthcare providers adjust their care for this group accordingly. Therefore, raising dental healthcare providers' awareness of the importance of understanding patients' perspectives and cultures is highly significant to ensure improving access to dental health services (17).

## 5 Limitations

The search was restricted to online sources, and only English-written papers were included in the study, which may have excluded papers from non-English-speaking nations. Additionally, the scoping review is a type of literature review that aims to map out the existing research on a particular topic. In accordance with JBI Scoping Review guidance, it is not mandatory to assess the quality of the evidence, which means that the findings may be influenced by the quality of the studies reviewed.

Another limitation of the studies reviewed is that they solely focused on adult refugees and excluded child refugees. Moreover, this scoping review has only included the refugee population while it does not cover anything about the asylum seekers who have not got the decision to stay legally in the country yet.

Most studies were qualitative in nature, which means that the findings may not be generalizable to larger populations. Another limitation is that the studies were conducted in different countries with varying healthcare systems and cultural contexts, which may limit the applicability of the findings to other settings.

## 6 Conclusion

The review acknowledges limitations the current literature, particularly in describing the characteristics of dental services accessed by refugees, and the assessment of effectiveness and impact of these services on quality of life of refugees living the host countries. The gaps on these areas provide valuable insights for future research. The barriers to access dental care are related to affordability, acceptability, accommodation, availability, awareness, and accessibility. Efforts are needed to improve access to dental services for refugees, including increasing awareness on understanding the healthcare system in the host country, promoting inter-professional collaborations that can lead to a more integrated health care, providing culturally sensitive care, and addressing financial and social barriers.

Further research is necessary to better understand some underexplored factors influencing access such as the importance of building trust relationships among dental practitioners and patients, and to develop practical solutions. It is also important to conduct research on accessing dental services among refugees in developing countries and refugee camps. Follow up research is needed to understand the characteristics of dental health services that refugees access in their host counties.
